# Chitosan activates NLRP3 inflammasome and cGAS-STING to suppress cancer progression through hexokinase 2 dissociation and mitochondrial dysfunction

**DOI:** 10.7150/thno.112009

**Published:** 2025-07-25

**Authors:** Lu Li, Liting You, Zhenfei Bi, Ziqi Zhang, Binwu Ying, Min Luo, Xiawei Wei

**Affiliations:** 1 Laboratory of Aging Research and Cancer Drug Target, Department of Biotherapy and Cancer Center, State Key Laboratory of Biotherapy, National Clinical Research Center for Geriatrics, West China Hospital, Sichuan University, Chengdu, Sichuan Province 610041, P. R. China.; 2 Clinical Trial Center, West China Hospital, Sichuan University, Chengdu, Sichuan Province 610041, P. R. China.; 3 Department of Laboratory Medicine, West China Hospital, Sichuan University, Chengdu, Sichuan Province 610041, P. R. China.

**Keywords:** chitosan, cancer, hexokinase 2, mitochondrial dysfunction, NLRP3 inflammasome

## Abstract

**Background:** Chitosan, a natural polysaccharide with known immunostimulatory potential, has shown promise in cancer therapy. However, its direct role in modulating antitumor immunity and the underlying mechanisms remain unclear. This study aimed to explore how unmodified chitosan influences tumor progression and immune responses through innate immune signaling pathways.

**Methods:** Murine tumor models (CT26, B16-F10) were used to evaluate the antitumor effects of chitosan in vivo. Flow cytometry and histological analyses assessed changes in immune cell infiltration. Primary macrophages and gene knockout models were used to investigate the molecular mechanisms, including inflammasome activation, mitochondrial function, and hexokinase 2 (HK2) location, via ELISA, western blotting, mitochondrial assays.

**Results:** Chitosan treatment suppressed tumor growth and metastasis, while promoting infiltration of neutrophils, monocytes, and activated T cells in the tumor microenvironment. Mechanistically, chitosan and its bioactive degradation product, N-acetylglucosamine (NAG), induced the dissociation of HK2 from mitochondria, triggering mitochondrial dysfunction, ROS overproduction, and mtDNA release. These signals jointly activated both the NLRP3 inflammasome and the cGAS-STING pathway. The antitumor effect of chitosan was attenuated in *Nlrp3*^-/-^ and *Sting*^-/-^ mice, confirming the essential roles of both pathways.

**Conclusions:** Chitosan orchestrates dual activation of NLRP3 and cGAS-STING signaling via HK2 dissociation and mitochondrial dysfunction, reprogramming the tumor immune microenvironment and enhancing antitumor immunity. These findings support chitosan's potential as a multifunctional immunoadjuvant for improving immunotherapy in resistant cancers.

## Introduction

Chitosan, a chitin-derived biopolymer of β-(1-4)-linked D-glucosamine (GAM) and N-acetyl-D-glucosamine (NAG) 1, exhibits antitumor 2, 3, antimicrobial 4, antidiabetic 5, wound healing 6, and antioxidant properties 7. Due to its numerous advantages such as non-toxicity, biocompatibility, and biodegradability, chitosan has been extensively explored in the field of biomedical applications, particularly in cancer therapy research 8. However, its widespread utilization is hindered by its insolubility in water and certain organic solvents. To overcome this limitation, various chitosan derivatives have been developed to retain their distinctive characteristics and expand their applicability. For example, Meng et al. reported the induction of potent immunogenic ferroptosis in hepatocellular carcinoma using dextran-chitosan hydrogels; furthermore, an increasing number of chitosan derivatives have shown efficacy in anti-cancer interventions and immune regulation 9. Nevertheless, the effects of unmodified chitosan on cancer development and anticancer immune responses remain unclear.

The immunomodulatory properties of chitosan have been extensively investigated. Previous studies have provided evidence supporting the notion that chitosan can stimulate the secretion of various cytokines and chemokines within innate immune cell populations, including macrophages, dendritic cells (DCs), and natural killer (NK) cells, thereby facilitating immune activation 10-12. Notably, Carroll et al. conducted experiments demonstrating that chitosan can enhance the generation of reactive oxygen species (ROS) and the release of mitochondrial DNA (mtDNA) within immune cells, thereby activating the cGAS-STING pathway, orchestrating type I interferon responses, and ultimately eliciting Th1 cell-mediated immune responses 13. Furthermore, chitosan has been shown to modulate both innate and adaptive immune responses through NLRP3 inflammasome activation and other associated molecular pathways 14, 15. However, current investigations are still exploring whether chitosan-induced activation of the NLRP3 inflammasome can confer anticancer effects along with its underlying immunomodulatory mechanisms.

In this study, we investigated the antitumor properties of unmodified chitosan by elucidating its role in activating the NLRP3 inflammasome. Furthermore, we presented empirical evidence highlighting chitosan's ability to facilitate the dissociation of mitochondrial HK2, leading to mitochondrial dysfunction and subsequent release of mitochondrial DNA (mtDNA) into the cytoplasm. This sequential cascade ultimately triggers activation of the STING pathway through which chitosan regulates anticancer immune responses.

## Results

### Chitosan suppresses cancer growth and metastasis in tumor models

**Figure 1:**
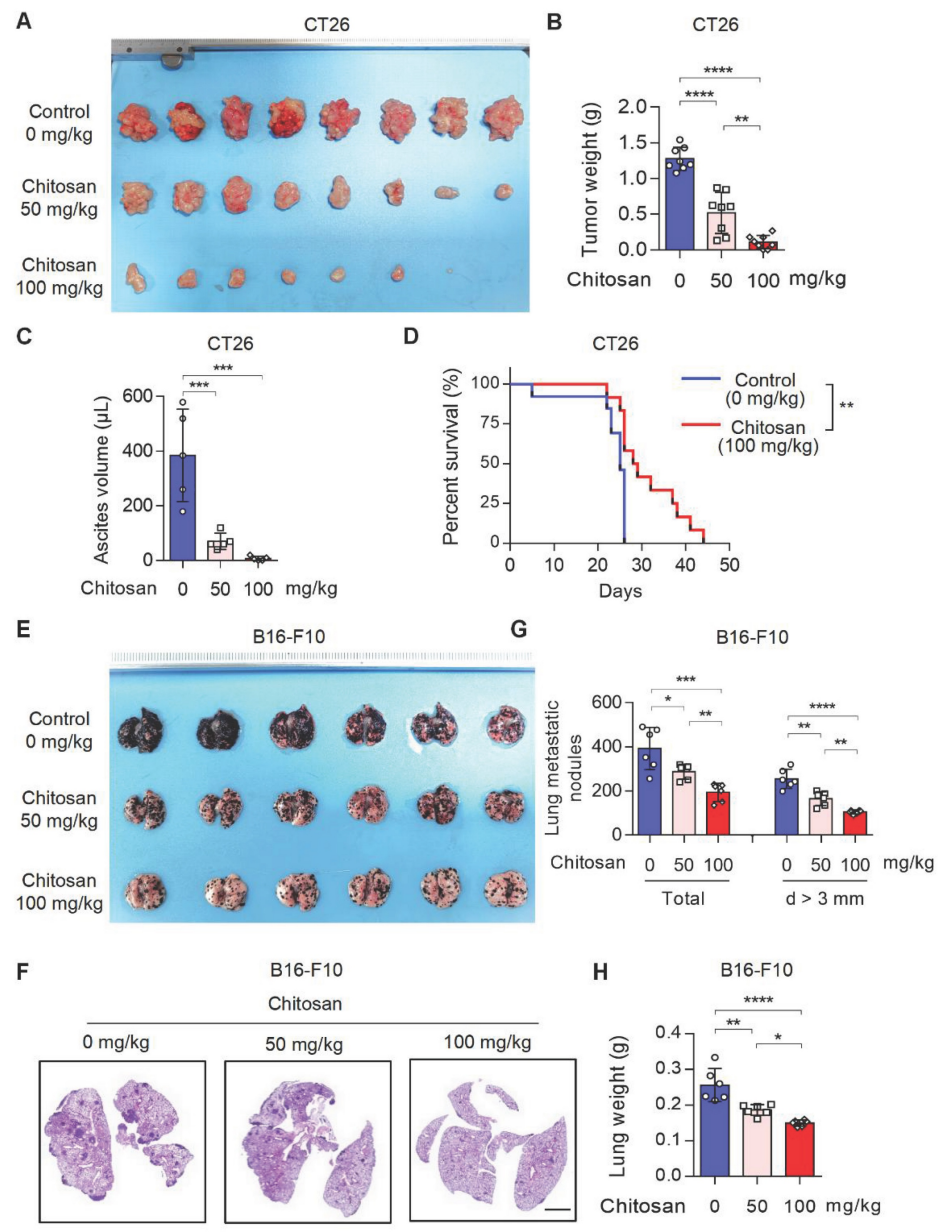
** Chitosan suppresses tumor growth and pulmonary metastasis. (A-C)** Chitosan suppresses tumor growth in CT26 peritoneal tumor model. CT26 cells (2 × 10^5^) were transplanted into the peritoneal cavity of mice to establish CT26 peritoneal tumor model. Mice were intraperitoneally administrated with 0, 50 or 100 mg/kg chitosan every three days and were sacrificed on day 15. Then peritoneal tumors were dissected and gross images were showed (A, n = 8 mice). Tumor weight (B, n = 8 mice) and ascites volume (C, n = 5 mice) were measured. **(D)** Chitosan prolonged the survival of tumor-bearing mice. Survival statistics of mice from CT26 (2 × 10^5^) peritoneal tumor model (Control: n = 13 mice, Chitosan: n = 12 mice). **(E-H)** Chitosan suppresses tumor metastasis in B16-F10 lung metastatic tumor model. B16-F10 cells (2 × 10^5^) were intravenously injected into mice to establish experimental pulmonary metastasis models (n = 6 mice). Mice were intraperitoneally administrated with 0, 50 or 100 mg/kg chitosan and sacrificed on day 15. Then pulmonary physiology was evaluated, including gross images (E) and H&E staining (F) of lungs. Scale bars represent 2 mm. Total number of metastatic nodules and the number of metastatic nodules with a diameter (d) greater than 3 mm were measured (n = 5 mice) (G). Lung weight was measured (n = 5 mice) (H). Data are represented as mean±SD. Statistical significance in (B, C, G, H) was determined by one-way ANOVA. Survival data in (D) were analyzed by log-rank test. ANOVA, analysis of variance; NS, not significant, *p < 0.05, ** *p* < 0.01, *** *p* < 0.001.

Chitosan and its derivatives have gained attention as potential molecular carriers with anti-tumor properties 16, 17. However, the precise role of unmodified chitosan in the progression and metastasis of various tumor types remains relatively unexplored. To address this knowledge gap, we conducted an investigation into the anti-tumor effects of unmodified chitosan using three distinct mouse tumor models: CT26 peritoneal tumor model, B16-F10 experimental lung metastasis model, and B16-F10 peritoneal tumor model (Supplemental Figure S1A). In the CT26 peritoneal tumor model, treatment with chitosan (0, 50, 100 mg/kg) resulted in a significant reduction in both tumor volume (Figure 1A and Supplemental Figure S1B) and tumor weight (Figure 1B). This was accompanied by a noticeable decline in carcinomatous ascites production (Figure 1C) and a substantial improvement in overall survival rates (Figure 1D). In the murine experimental pulmonary metastasis model established by intravenous injection of B16-F10 cells, we observed a dose-dependent decrease in tumor metastasis among chitosan-treated groups (Figure 1E-F). Furthermore, there was a considerable decrease in the total number of metastatic nodules and nodules with a diameter greater than 3 mm in the lungs of chitosan-treated mice (Figure 1G). Additionally, lung weight decreased proportionally to the administered doses of chitosan treatment (Figure 1H). Similar anti-tumor effects were consistently observed with higher doses of chitosan treatment in the B16-F10 peritoneal tumor model (Supplemental Figure S1C). Simultaneously, we demonstrated the absence of significant toxicity following chitosan treatment through histological staining and functional biochemical analysis of the mouse heart, liver, spleen, lung, and kidney (Supplemental Figure S2).

### Chitosan activates tumor immune microenvironment with increased infiltration of neutrophil and monocytes

**Figure 2:**
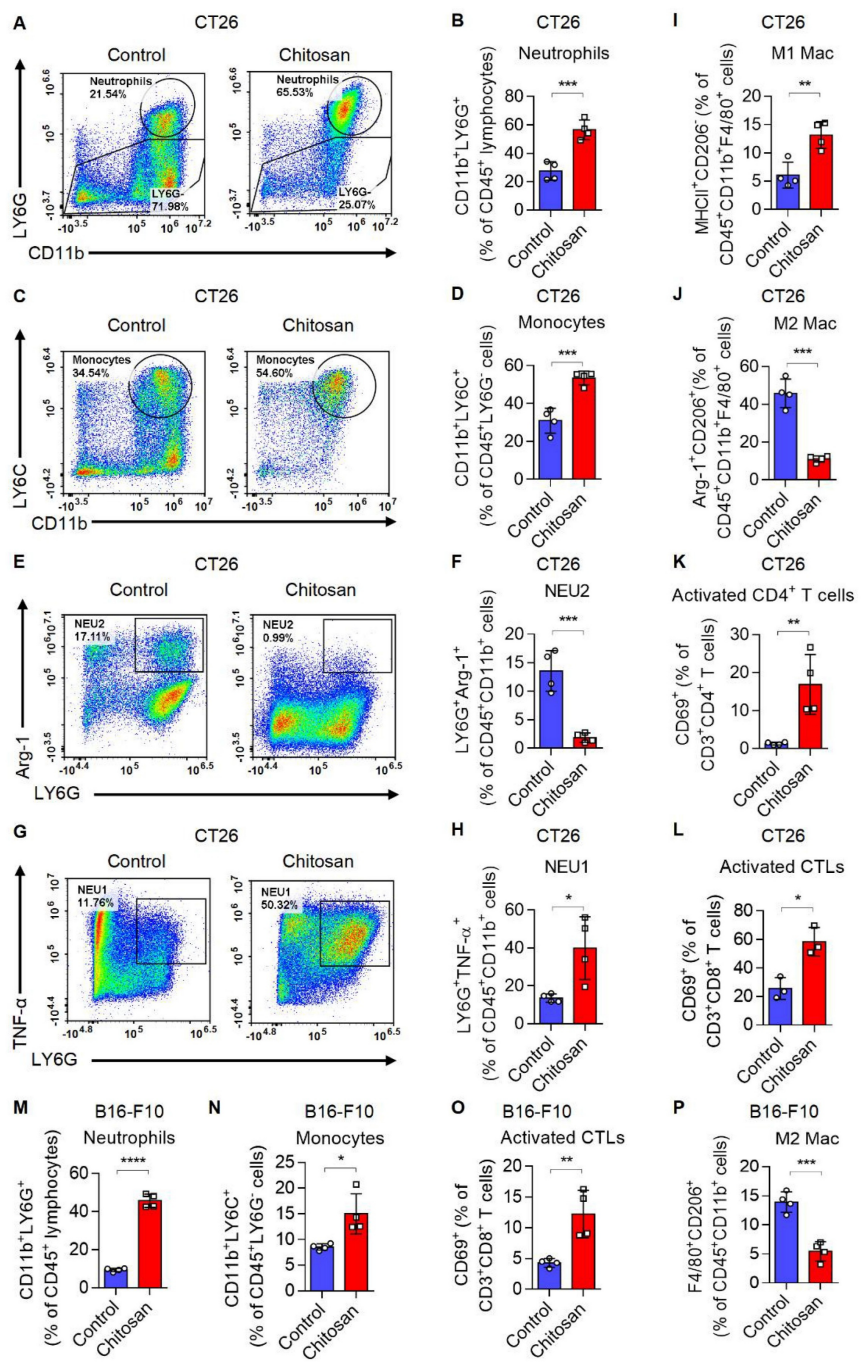
**Chitosan transforms tumor immune microenvironment and increases infiltration of neutrophils and monocytes. (A-L)** Chitosan transforms tumor immune microenvironment in CT26 peritoneal tumor model. CT26 cells (2 × 10^5^) were transplanted into the peritoneal cavity of mice to establish CT26 peritoneal tumor model. Mice were intraperitoneally administrated with 0 or 100 mg/kg chitosan every three days and sacrificed on day 15 to collect the tumors. Then the single-cell suspension of tumors was prepared and subjected to FCM analysis. (A-B) Representative scatterplots of the gated neutrophils (CD45^+^ CD11b^+^ LY6G^+^) are shown in (A) and quantified in (B) (n = 4 mice). (C-D) Representative scatterplots of the gated monocytes (CD45^+^ CD11b^+^ LY6G^-^ LY6C^+^) are shown in (C) and quantified in (D) (n = 4 mice). (E-F) Representative scatterplots of the gated N2 neutrophils (NEU2, CD45^+^ CD11b^+^ LY6G^+^ Arg-1^+^) are shown in (E) and quantified in (F) (n = 4 mice). (G-H) Representative scatterplots of the gated N1 neutrophils (NEU1, CD45^+^ CD11b^+^ LY6G^+^ TNF-α^+^) are shown in (G) and quantified in (H) (n = 4 mice). (I-L) The percentages of M1 macrophages (M1 Mac, CD45^+^ CD11b^+^ F4/80^+^ MHCII^+^ CD206^-^), M2 Macrophages (M2 Mac, CD45^+^ CD11b^+^ F4/80^+^ Arg-1^+^ CD206^+^), activated CD4^+^ T cells (CD3^+^ CD4^+^ CD69^+^), and activated CTLs (CD3^+^ CD8^+^ CD69^+^), are quantified. **(M-P)** Chitosan transforms tumor immune microenvironment in B16-F10 experimental pulmonary metastasis models. B16-F10 cells (2×10^5^) were intravenously injected into mice to establish experimental pulmonary metastasis models. Mice were administrated with 0 or 100 mg/kg chitosan and sacrificed on day 15 to collect the lungs. Then the single-cell suspension of lungs and metastatic tumors was prepared and subjected to FCM analysis. The percentages of neutrophils (CD45^+^ CD11b^+^ LY6G^+^), monocytes (CD45^+^ CD11b^+^ LY6G^-^ LY6C^+^), activated CTLs (CD3^+^ CD8^+^ CD69^+^), and M2 Macrophages (CD45^+^ CD11b^+^ F4/80^+^ CD206^+^) are quantified (n = 4 mice). Data are represented as mean±SD. Statistical significance in (B, D, F, H, I-P) was determined by a two-sided unpaired t-test. * *p* < 0.05, ** *p* < 0.01, *** *p* < 0.001, **** *p* < 0.0001.

Chitosan has been acknowledged for its multifaceted immunostimulatory properties 18. To gain deeper insights into the immunomodulatory mechanisms underlying chitosan's anti-tumor effects, we conducted a comprehensive analysis of alterations within the tumor immune microenvironment in mice treated with chitosan. Firstly, we investigated how chitosan modulates the TME in CT26 peritoneal tumor model. We observed a significant increase in the population of CD11b^+^ Ly6G^+^ neutrophils (Figure 2A-B) and CD11b^+^ Ly6C^+^ Ly6G^-^ monocytes (Figure 2C-D) following chitosan treatment, indicating an activated inflammatory immune response. Given the dual roles of monocytes/macrophages and neutrophils in tumors, we subsequently analyzed the polarization status of these cells within the chitosan-induced TME. The results demonstrated a significant increase in N1 neutrophils (NEU1, TNF-α^+^) and a marked reduction in N2 neutrophils (NEU2, Arg-1^+^) in the TME following chitosan treatment (Figure 2E-H). Concurrently, we observed that chitosan induced an elevation of M1 macrophages (M1 Mac, MHCII^+^) while reducing the population of M2 macrophages (M2 Mac, CD206^+^Arg-1^+^) (Figure 2I-J, Supplemental Figure S3A-B). These findings showed that chitosan reprograms myeloid cells in the TME toward anti-tumor phenotypes. Interestingly, there was a marked and statistically significant increase in the percentage of CD8^+^ cytotoxic T lymphocytes (CTLs), activated CTLs (CD8^+^ CD69^+^) and activated CD4^+^ T cells (CD4^+^ CD69^+^) (Figure 2K-L, Supplemental Figure S3C).

These findings suggest that chitosan enhances immune-activated cells while reducing immune-suppressed cells. Furthermore, in the B16-F10 lung metastasis model, lung tissues were collected after chitosan treatment and single-cell suspensions were prepared for subsequent flow cytometric analysis to meticulously characterize each immune cell type. We observed elevated percentages of CD11b^+^ Ly6G^+^ neutrophils, CD11b^+^ Ly6G^-^ Ly6C^+^ monocytes, CD8^+^ CTLs, and activated CTLs (CD8^+^ CD69^+^), as well as decreased percentage of M2 macrophages upon chitosan treatment (Figure 2M-P, Supplemental Figure S3D); indicative of an activation of the tumor immune microenvironment. Consistently, in B16-F10 peritoneal tumor model, chitosan also demonstrated its ability to transform the tumor immune environment (Supplemental Figure S3E-H). Our observations highlight the crucial role played by chitosan in modulating the landscape of the tumor immune system; however, the precise underlying regulatory mechanisms require further investigation.

### Chitosan elicits antitumor effects and modulates the tumor immune environment through the activation of the NLRP3 inflammasome

**Figure 3:**
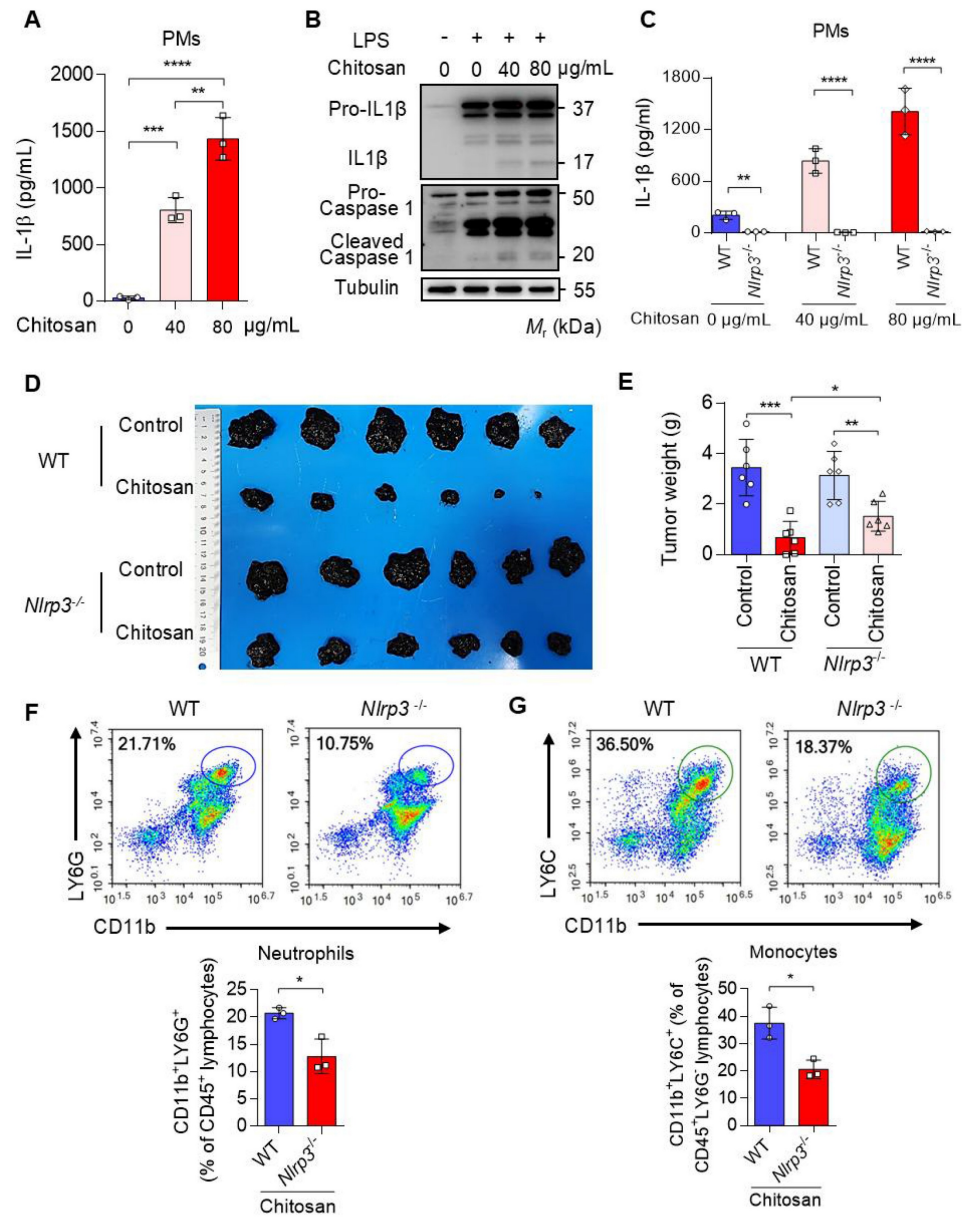
** The anti-tumor effect of chitosan depends on the activated NLRP3 inflammasome. (A-B)** Chitosan promotes the secretion of IL-1β in peritoneal macrophages (PMs). LPS-pretreated PMs from wild-type (WT) mice were stimulated with 0, 40 or 80 μg/mL chitosan for 6 hours. Then the IL-1β levels in the supernatants were detected by ELISA (n = 3 biologically independent samples) (A). And the pro- IL-1β, IL-1β, pro-caspase 1 and cleaved caspase 1 protein in PMs were detected by WB (B). **(C)** *Nlrp3* deficiency inhibits the secretion of IL-1β in PMs after chitosan stimulation. LPS-pretreated PMs from WT or *Nlrp3*^-/-^ mice were stimulated with 0, 40 or 80 μg/mL chitosan for 6 hours. Then the IL-1β levels in the supernatants were detected by ELISA (n = 3 biologically independent samples). **(D-E)** The anti-tumor effect of chitosan depends on the activated NLRP3. B16-F10 cells (2 × 10^5^) were transplanted into the peritoneal cavity of WT or *Nlrp3*^-/-^ mice to establish B16-F10 peritoneal tumor model. Mice were intraperitoneally administrated with 0 or 100 mg/kg chitosan every three days and sacrificed on day 15. Then peritoneal tumors were dissected and gross images were showed (D). Tumor weight (E) was measured (n = 6 mice). **(F-G)** *Nlrp3* deficiency inhibits the infiltration of neutrophils and monocytes. Then the single-cell suspension of above tumors was prepared and subjected to FCM analysis (n = 3 mice). (F) Representative scatterplots of the gated neutrophils (CD45^+^ CD11b^+^ LY6G^+^) are shown in the upper panel and quantified in the lower panel (n = 3 mice). (G) Representative scatterplots of the gated monocytes (CD45^+^ CD11b^+^ LY6G^-^ LY6C^+^) are shown in the upper panel and quantified in the lower panel (n = 3 mice). Data are represented as mean±SD. Statistical significance in (A, E) was determined by one-way ANOVA. Statistical significance in (C, F, G) was determined by a two-sided unpaired t-test. * *p* < 0.05, ** *p* < 0.01, *** *p* < 0.001, **** *p* < 0.0001.

Chitosan, a well-documented NLRP3 agonist renowned for its ability to stimulate the NLRP3 inflammasome and induce IL-1β release 19, was validated for its capacity to induce IL-1β secretion in peritoneal macrophages (PMs) (Figure 3A-B). Notably, deletion of *Nlrp3* impeded the upregulation of IL-1β expression induced by chitosan (Figure 3C). Furthermore, we observed a dose-dependent increase in IL-1β secretion with escalating concentrations of chitosan (Figure 3A, C), accompanied by elevated levels of cleaved caspase-1 responsible for processing pre-IL-1β into its mature form (Figure 3B). However, it is important to acknowledge that the role of NLRP3 inflammasome in tumor development is dual 20, 21. Therefore, an investigation was conducted to determine whether the antitumor efficacy of chitosan relies on the activation of NLRP3 inflammasome. To compare the antitumor effects of chitosan between wild-type (WT) mice and *Nlrp3* knockout (*Nlrp3*^-/-^) mice, experiments were performed using B16-F10 peritoneal tumors. Our findings unequivocally demonstrate that chitosan exerts a significant inhibitory effect on tumor growth in wild-type (WT) mice; however, this antitumor activity is conspicuously diminished in *Nlrp3*^-/-^ mice (Figure 3D). In comparison to their chitosan-treated WT counterparts, *Nlrp3*^-/-^ mice exhibit notably larger tumor volumes (Figure 3D) and tumor weights (Figure 3E), thereby underscoring the pivotal role of NLRP3 in mediating the antitumor effect of chitosan. To investigate how *Nlrp3* deficiency affects the chitosan-stimulated tumor microenvironment (TME), we further analyze the percentages of immune cell populations in *Nlrp3*^-/-^ mice compared with their WT counterparts, particularly neutrophils and monocytes responsible for IL-1β inflammation. Our investigation reveals a noteworthy reduction in the proportion of infiltrating neutrophils (CD11b^+^ LY6G^+^) within the TME of *Nlrp3*^-/-^ mice, as well as monocytes (CD11b^+^ Ly6G^-^Ly6C^+^) (Figure 3F-G), contrasting sharply with those observed in the WT group. These findings imply that the antitumor efficacy of chitosan may be attributed to activation of the NLRP3 inflammasome, consequently promoting infiltration of neutrophils and monocytes into tumor tissue. A concentration of 80 μg/mL of chitosan was found to significantly enhance the expression and secretion of cytokines by macrophages compared to a concentration of 40 μg/mL. Therefore, the dose of 80 μg/mL was selected for further investigation into the underlying mechanism.

### The bioactive anti-tumor component of chitosan is N-acetylglucosamine (NAG)

**Figure 4:**
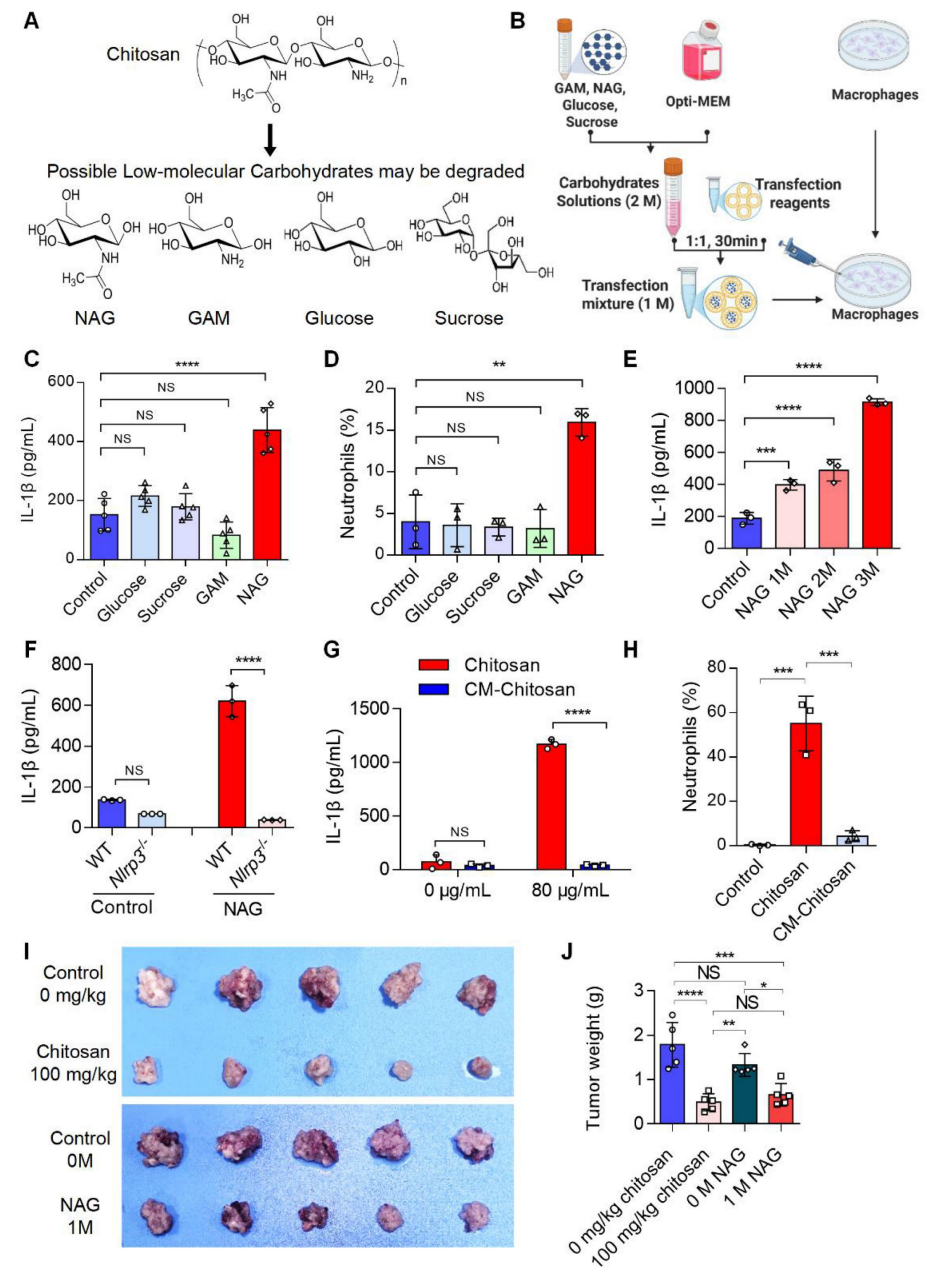
** The active anti-tumor component of chitosan is N-acetylglucosamine (NAG). (A)** The chemical formulas of low-molecular carbohydrates may be degraded from chitosan. **(B)** Experimental workflow of the in vitro macrophage transfection assay. **(C)** NAG increases IL-1β secretion of PMs. LPS-pretreated PMs isolated from WT mice were stimulated with glucose (1 M), sucrose (1 M), GAM (1 M) and NAG (1 M) for 6 hours, then IL-1β levels in the supernatants were detected by ELISA (n = 5 biologically independent samples). Control: liposome. **(D)** NAG increases the percentage of neutrophils infiltrated in peritoneal of WT mice. Glucose (1 M), sucrose (1 M), GAM (1 M) and NAG (1 M) were injected into the peritoneal of mice for 4 hours. Control: liposome. Then peritoneal lavage cells were subjected to FCM analysis to quantify the percentages of neutrophils. **(E)** NAG increases IL-1β levels in PMs in a dose-dependent manner. LPS-pretreated PMs were stimulated with 1, 2, or 3M NAG for 6 hours, then IL-1β levels in the supernatants were detected by ELISA (n = 3 biologically independent samples). **(F)** NAG induced increase of IL1β depends on the activated NLRP3. LPS-pretreated PMs isolated from WT or *Nlrp3*^-/-^ mice were stimulated with or without NAG for 6 hours, then IL-1β levels in the supernatants were detected by ELISA (n = 3 biologically independent samples). Control: liposome. **(G-H)** Carboxymethyl modification in NAG of chitosan reduces IL-1β levels and inhibits neutrophil infiltration. (G) LPS-pretreated PMs isolated from WT mice were stimulated with Chitosan or Carboxymethyl-chitosan (CM-Chitosan) for 6 hours, then IL-1β levels in the supernatants were detected by ELISA (n = 3 biologically independent samples). (H) Chitosan or CM-Chitosan were injected into the peritoneal of mice for 4 hours. Then peritoneal lavage cells were subjected to FCM analysis to quantify the percentages of neutrophils. **(I-J)** NAG suppresses tumor growth in CT26 peritoneal tumor model. CT26 cells (2 × 10^5^) were transplanted into the peritoneal cavity of wild-type (WT) mice to establish CT26 peritoneal tumor model. Mice were intraperitoneally administrated with 0 mg/kg chitosan (200 μL/mice), 100mg/kg chitosan (200 μL/mice), 0 M NAG (100 μL/mice), or 1 M NAG (100 μL/mice) every three days, and sacrificed on day 15. Then peritoneal tumors were dissected. Tumor gross images were showed (I, n = 5 mice) and tumor weight (J, n = 5 mice) were measured. Data are represented as mean±SD. Statistical significance in (C, D, E, H, J) was determined by one-way ANOVA. Statistical significance in (F, G) was determined by a two-sided unpaired t-test. NS, not significant, * *p* < 0.05, ** *p* < 0.01, *** *p* < 0.001, **** *p* < 0.0001.

Chitosan may degrade into low-molecular-weight carbohydrates such as N-acetylglucosamine (NAG), glucosamine (GAM), glucose, and sucrose (Figure 4A). To identify the active component responsible for chitosan's antitumor effects, we delivered these carbohydrates (1 M) into macrophages via liposome transfection (Figure 4B). Our data revealed that NAG, but not other sugars, dose-dependently enhanced IL-1β production in macrophages and promoted neutrophil recruitment (Figure 4C-E). Notably, similar to chitosan, NAG-induced IL-1β production in macrophages was critically dependent on NLRP3 activation (Figure 4F), suggesting NAG as a key immune effector component of chitosan. To further validate the pivotal role of NAG in the antitumor activity of chitosan, we administered N-carboxymethyl chitosan (CM-CS), a derivative created by carboxymethylation of NAG residues in chitosan. CM-CS failed to effectively induce IL-1β production or neutrophil recruitment (Figure 4G, H), confirming the structural specificity of NAG in driving immunomodulatory responses. Subsequent in vivo experiments revealed that NAG recapitulated chitosan's antitumor properties by significantly suppressing tumor growth and reducing tumor burden (Figure 4I, J). However, in vitro experiments revealed that chitosan exhibited direct cytotoxic effects on tumor cells, while NAG showed no significant direct tumor-killing activity, underscoring its exclusive role in immune-mediated antitumor responses (Supplemental Figure S4). Collectively, these findings establish NAG as the pivotal bioactive component underlying chitosan-induced antitumor immunity through NLRP3-dependent macrophage activation and subsequent immune cell recruitment.

### Chitosan activates NLRP3 inflammasome and cGAS-STING through the dissociation of mitochondrial HK2 and induction of mitochondrial dysfunction

**Figure 5:**
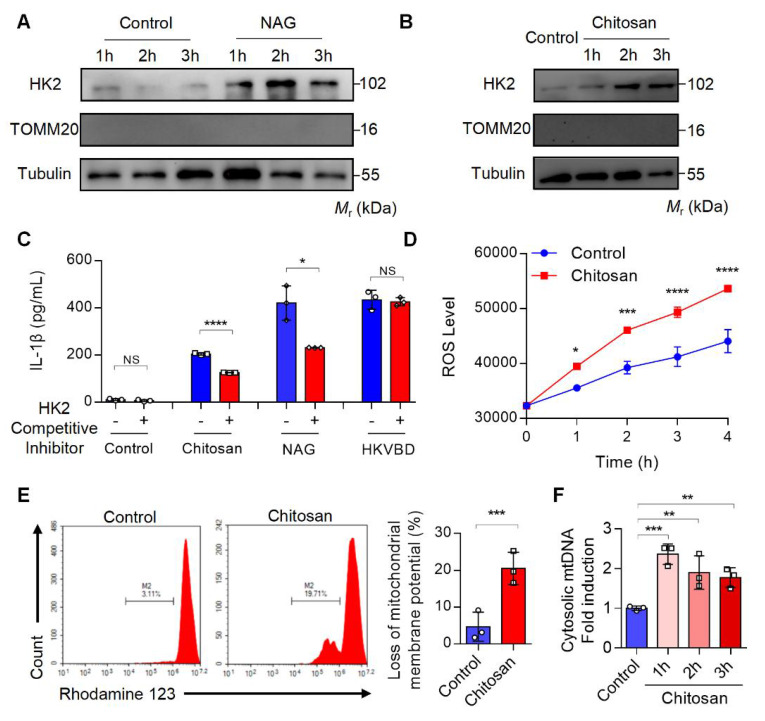
**Chitosan activates the NLRP3 inflammasome through the dissociation of mitochondrial HK2 and induction of mitochondrial dysfunction. (A-B)** Chitosan activates NLRP3 inflammasome through the dissociation of mitochondrial HK2. LPS-pretreated PMs from WT mice were stimulated with NAG (1 M) (A) or chitosan (80 μg/mL) (B) for indicated time. Then the cytoplasmic protein was extracted and HK2 levels were detected by WB. TOMM20 (Translocase of Outer Mitochondrial Membrane 20) was used as a mitochondrial marker. Tubulin was used as a cytoplasmic internal reference protein. **(C)** HK2 competitive inhibitor reduces the increased IL-1β levels induced by chitosan and NAG. HK2 competitive inhibitor, glucose, was used to treat control, chitosan, NAG and HKVBD stimulated cells. The IL-1β levels in the supernatants were detected by ELISA (n = 3 biologically independent samples). **(D-F)** Chitosan induces mitochondrial dysfunction of PMs. LPS-pretreated PMs were stimulated with or without 80 μg/mL chitosan. ROS levels were monitored (n = 3 biologically independent samples) (D) and mitochondrial membrane potential was detected by Rhodamine 123 (RH123) FCM analysis (n = 3 biologically independent samples) (E). The PMs were subjected to qPCR analysis to detect the relative mtDNA levels, *mtCOX1* as an internal reference (n = 3 biologically independent samples) (F). Data are represented as mean±SD. Statistical significance in (C, E) was determined by a two-sided unpaired t-test. Statistical significance in (D) was determined by two-way ANOVA. Statistical significance in (F) was determined by one-way ANOVA. NS, not significant, * *p* < 0.05, ** *p* < 0.01, *** *p* < 0.001, **** *p* < 0.0001

Previous studies have demonstrated that NAG binds to the glucose-binding site of hexokinase 2 (HK2), a mitochondrial outer membrane protein, triggering HK2 detachment from mitochondria and its translocation to the cytoplasm^22^. This HK2 relocation enhances mitochondrial membrane permeability, resulting in mitochondrial dysfunction, excessive reactive oxygen species (ROS) generation, mitochondrial DNA (mtDNA) release, and subsequent inflammatory responses^22^. To investigate whether chitosan similarly induces HK2 mitochondrial-cytoplasmic relocation, we stimulated macrophages with NAG (1 M) or chitosan (80 μg/mL), followed by subcellular fractionation to assess HK2 levels in mitochondrial and cytosolic compartments. Both treatments significantly increased cytosolic HK2 (Figure 5A-B) while reducing mitochondrial HK2 (Supplemental Figure S5A-B), paralleled by elevated IL-1β production in macrophages (Figure 5C). Strikingly, competitive inhibition of NAG-HK2 binding with glucose markedly attenuated chitosan- and NAG-induced IL-1β secretion (Figure 5C), confirming the critical role of HK2 displacement in their pro-inflammatory activity.

Chitosan and its bioactive degradation product NAG may disrupt the interaction between HK2 and mitochondria, leading to mitochondrial dysfunction and amplifying inflammation through ROS- and mtDNA- driven pathways 23 24. To validate this mechanism, we measured mitochondrial membrane potential, ROS levels, and mtDNA content in chitosan-treated macrophages. Compared to untreated controls, chitosan stimulation triggered a significant increase in intracellular ROS (Figure 5D), severe mitochondrial damage marked by a pronounced loss of membrane potential (Figure 5E, Supplemental Figure S5C), and elevated mtDNA copy numbers in the cytoplasm (Figure 5F). To establish the causal sequence linking HK2 dissociation to mitochondrial dysfunction in NAG-mediated effects, we synthesized HK2-VDAC-binding domain mimic (HKVBD), a peptide mimicking HK2 displacement from mitochondria, and delivered into macrophages. HKVBD treatment recapitulated key features of NAG/chitosan-induced pathology, including significant mitochondrial membrane potential collapse and enhanced IL-1β production (Supplemental Figure S5D-E). Crucially, unlike NAG/chitosan-driven IL-1β elevation, HKVBD-triggered IL-1β secretion remained unaffected by glucose-mediated competitive inhibition (Figure 5C). These data mechanistically confirm that HK2 displacement constitutes the essential first step in this pathway. Chitosan, via NAG-mediated HK2 displacement, compromises mitochondrial integrity, thereby activating pro-inflammatory cascades through ROS overproduction and mtDNA release.

**Figure 6:**
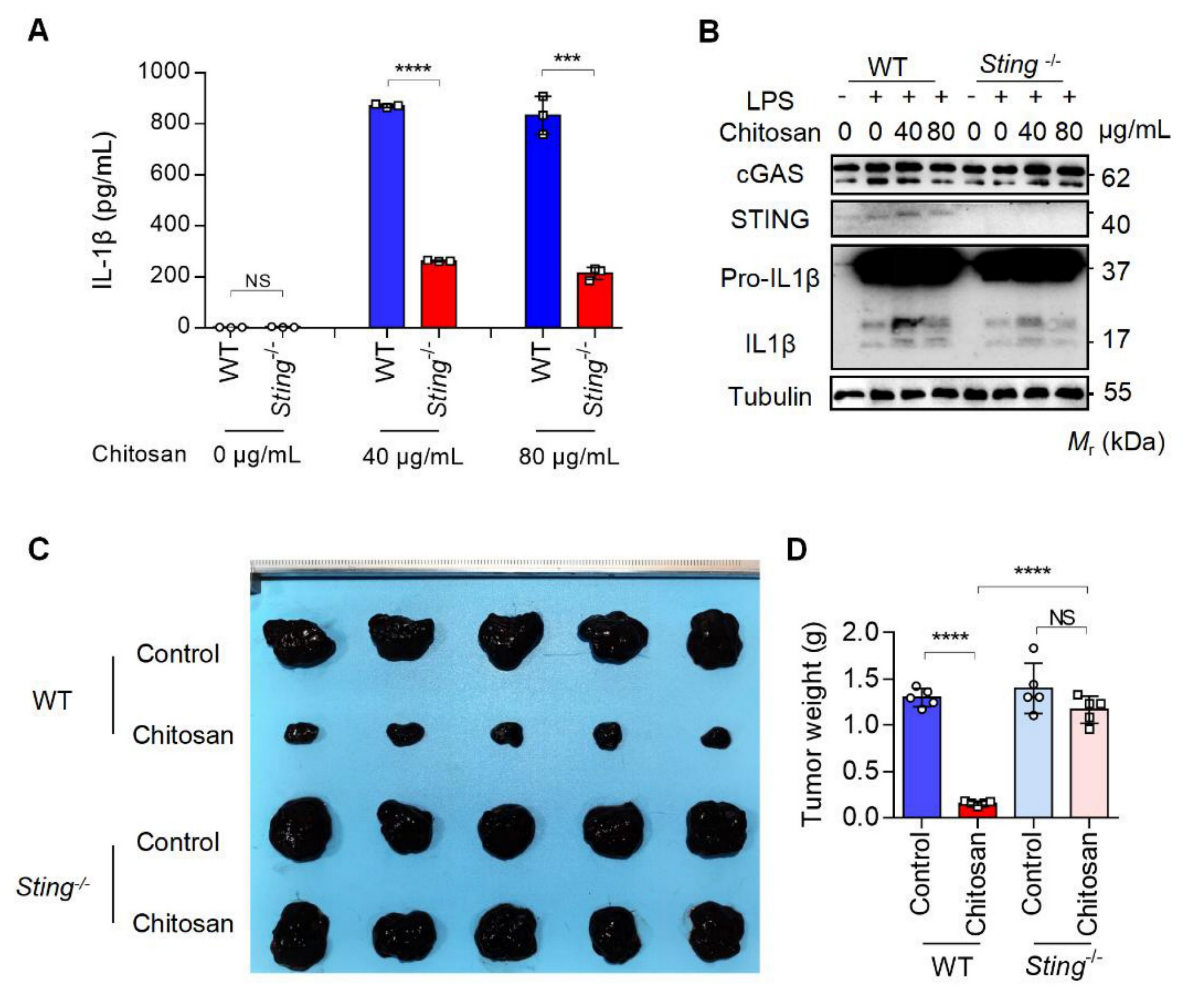
** The anti-tumor and immunomodulatory effects of chitosan also depend on STING. (A-B)** LPS-pretreated PMs from WT or *Sting*^-/-^ mice were stimulated with 0, 40, or 80 μg/mL chitosan for 6 hours. The IL-1β levels in the supernatants were detected by ELISA (n = 3 biologically independent samples) (A). And the pro-IL-1β, IL-1β, cGAS, and STING protein in PMs were detected by WB (B). **(C-D)** The anti-tumor effect of chitosan depends on STING. B16-F10 cells (2 × 10^5^) were transplanted into the peritoneal cavity of WT or *Sting*^-/-^ mice to establish B16-F10 peritoneal tumor model. Mice were intraperitoneally administrated with 0 or 100 mg/kg chitosan every three days and sacrificed on day 15. Then peritoneal tumors were dissected and gross images were showed (C). Tumor weight (D) was measured (n = 5 mice). Data are represented as mean ± SD. Statistical significance in (A) was determined by a two-sided unpaired t-test. Statistical significance in (D) was determined by one-way ANOVA. NS, not significant, *** *p* < 0.001, **** *p* < 0.0001.

**Figure 7:**
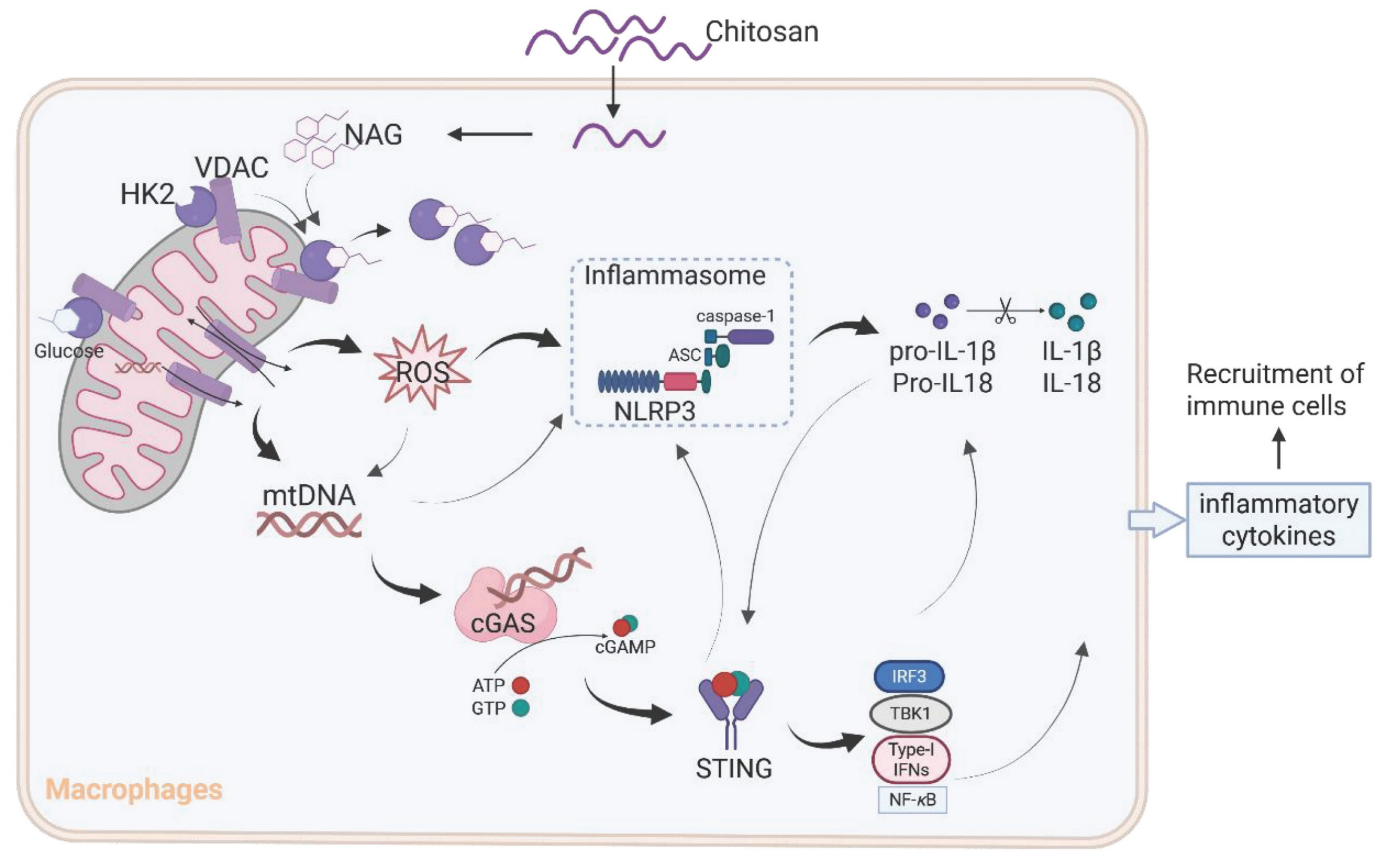
** A schematic diagram of the mechanism by which chitosan and its bioactive degradation product NAG exert immunomodulatory and antitumor effects.** Chitosan and NAG induce HK2 dissociation from mitochondria by occupying the glucose binding site, destabilizing the HK2-VDAC complex and impairing mitochondrial integrity. This disruption drives ROS overproduction and mtDNA release, which synergistically activate the NLRP3 inflammasome to promote IL-1β secretion. Concurrently, cytosolic mtDNA engages the cGAS-STING pathway, creating cross-amplification with NLRP3 signaling, while cooperatively enhancing pro-inflammatory cytokine production. This dual activation network amplifies immune cell recruitment and effector functions, ultimately orchestrating the observed antitumor effects.

The results from Figure 3D and 3E reveal that while NLRP3 deficiency markedly attenuated chitosan's antitumor efficacy, residual tumor reduction persisted, and crucially, ROS inhibition via NAC failed to suppress chitosan-induced IL-1β production (Supplemental Figure S5F), collectively suggesting complementary mechanisms beyond NLRP3. Given that mtDNA release can activate the cGAS-STING pathway and our observation of chitosan-induced elevation in STING-associated cytokines such as IL-6 and TNF-α alongside IL-1β (Supplemental Figure S5G), we hypothesized cGAS-STING signaling as a complementary axis in chitosan-mediated antitumor immunity. To test this, we employed STING-knockout (*Sting*^-/-^) models in vitro and in vivo. Chitosan-stimulated *Sting*^-/-^ macrophages exhibited markedly reduced IL-1β production and secretion compared to wild-type (WT) controls (Figure 6A-B). In vivo, *Sting*^-/-^ mice bearing tumors showed significantly increased tumor volumes and weights relative to chitosan-treated WT counterparts (Figure 6C-D), confirming STING's critical role in amplifying chitosan's antitumor effects. These findings collectively reveal a dual-pathway mechanism wherein chitosan engages both NLRP3-dependent inflammasome activation and cGAS-STING-driven cytokine signaling to orchestrate antitumor immune responses, with STING pathway activation compensating for NLRP3 loss to sustain partial therapeutic efficacy (Figure 7).

## Discussion

Although the immunomodulatory potential of chitosan and its derivatives has been previously documented 25-28, controversies persist regarding their specific immune effects and their molecular mechanisms 29, 30. Our study provides compelling evidence for a unified mechanism (Figure 7): chitosan and its bioactive degradation product NAG induce HK2 dissociation from mitochondria, destabilizing the HK2-VDAC complex and impairing mitochondrial integrity. This disruption drives ROS overproduction and mtDNA release, which synergistically activate the NLRP3 inflammasome to promote IL-1β secretion. Concurrently, cytosolic mtDNA engages the cGAS-STING pathway, creating cross-amplification with NLRP3 signaling and compensating for NLRP3 deficiencies, while cooperatively enhancing pro-inflammatory cytokine production (IL-1β, IL-6, TNF-α). This dual activation network amplifies immune cell recruitment and effector functions, ultimately orchestrating the observed antitumor effects.

While chitosan's therapeutic effects and mechanism are increasingly recognized, critical questions persist regarding its molecular weight-dependent bioactivity and detailed metabolic processing. Chitosan, a natural polysaccharide, exhibits molecular weight-dependent pharmacological behaviors, as exemplified by Maeda et al., who demonstrated that high-molecular-weight chitosan (650 kDa) showed limited antitumor efficacy, whereas low-molecular-weight variants (e.g., ~50 kDa, as used in this study) more effectively suppressed tumor growth 31 -a dichotomy likely tied to differential cellular uptake, degradation kinetics, and immune receptor interactions. Although our work identifies NAG as the primary bioactive component mediating chitosan's immunomodulatory and antitumor effects via mitochondrial HK2 dissociation and NLRP3/cGAS-STING crosstalk, uncertainties remain about the metabolic equivalence between chitosan and their degradation products (e.g., NAG, glucosamine, glucose, sucrose). Specifically, the stoichiometric relationship between administered chitosan doses and bioactive NAG concentrations in vivo remains undefined, raising questions about optimal dosing regimens and bioavailability. Future studies must delineate chitosan's enzymatic degradation pathways, tissue-specific metabolite profiles, and temporal dynamics of NAG release to bridge the gap between polymer administration and bioactive fragment availability, thereby refining chitosan-based therapeutic strategies for precision oncology applications.

Our findings reveal that chitosan orchestrates a profound immunomodulatory reprogramming of the tumor microenvironment (TME) by driving functional polarization of both neutrophils and macrophages toward antitumor phenotypes. Chitosan treatment significantly increased N1-polarized neutrophils (TNF-α⁺) while suppressing protumor N2 neutrophils (Arg-1⁺), concurrently skewing macrophages from immunosuppressive M2 (CD206⁺Arg-1⁺) to immunostimulatory M1 (MHCII⁺) populations—a dual shift that collectively converts the TME from immune-tolerant to immune-active. This polarization aligns with chitosan's previously demonstrated ability to trigger mitochondrial stress-induced mtDNA release and cGAS-STING/NLRP3 activation, as TNF-α (N1 marker) and MHCII (M1 marker) are hallmark outputs of these pathways. Notably, the coordinated reduction in Arg-1⁺ populations (shared by N2 neutrophils and M2 macrophages) suggests chitosan disrupts immunosuppressive arginase-mediated pathways, while the TNF-α/MHCII axis likely enhances antigen presentation and cytotoxic immune cell recruitment, mechanistically linking myeloid cell reprogramming to the observed tumor suppression.

While our study demonstrates that chitosan activates the NLRP3 inflammasome via HK2 dissociation and mitochondrial dysfunction to suppress tumor progression, we acknowledge the need to contextualize these findings within the broader complexity of NLRP3's dual roles in cancer 20, 21. The NLRP3 inflammasome is a double-edged sword: while its acute activation can drive anti-tumor immunity by promoting IL-1β/IL-18 secretion and immunogenic cell death, chronic or dysregulated NLRP3 signaling has been implicated in tumor-promoting inflammation, immunosuppressive microenvironment remodeling 32, and metastasis in certain contexts. Our work focuses on chitosan's short-term immunostimulatory effects in immunocompetent murine models (CT26, B16-F10), but we recognize that its therapeutic window may vary depending on tumor type, stage, and host immune status. For instance, in tumors with pre-existing inflammatory microenvironments, sustained NLRP3 activation by chitosan could theoretically exacerbate protumorigenic cytokine cascades (e.g., IL-1β-driven angiogenesis). To address this, future studies should evaluate chitosan in clinically relevant models, such as patient-derived xenografts (PDXs), while monitoring long-term toxicity and tumor recurrence. Additionally, combining chitosan with checkpoint inhibitors (e.g., anti-PD-1) may help mitigate potential risks of immunopathology while amplifying therapeutic synergy—a strategy supported by recent work showing NLRP3-driven inflammation can enhance PD-1 blockade efficacy in “cold” tumors 33.

Furthermore, there are some shortcomings in our study. Mechanistically, while our data link chitosan to HK2-VDAC dissociation and mtDNA release, further validation is required to dissect upstream regulators (e.g., chitosan's interaction with hexokinase isoforms beyond HK2) and downstream crosstalk with other inflammasomes (e.g., AIM2, NLRC4) or DNA sensors (e.g., cGAS). Finally, while chitosan represents a promising NLRP3-targeted immunomodulator, its translation will require careful optimization of dosing regimens, combinatorial strategies, and patient stratification to balance anti-tumor efficacy with inflammatory toxicity-a challenge reflective of the broader inflammasome therapeutics field.

## Materials and Methods

### Cells and animals

The mouse melanoma cell line (B16-F10) and mouse colon cancer cell line (CT26) were obtained from the American Type Culture Collection (ATCC). B16-F10 and CT26 cells were cultured in Dulbecco's modified Eagle's medium (DMEM) (Gibco, USA), supplemented with 10% fetal bovine serum (Gibco, USA), penicillin (100 U/ml), and streptomycin (100 μg/ml). All cells were maintained at 37 °C in a humidified atmosphere containing 5% CO_2_.

Primary peritoneal macrophages (PMs) were used for in vitro studies. A slow introduction of 5 mL of PBS into the abdominal cavity of mice was followed by gentle agitation to ensure proper contact between the PBS and the abdominal cavity. Subsequently, the PBS was aspirated using a syringe, repeating this process. The resulting cell suspension underwent centrifugation at 1500 rpm for 3 minutes, followed by two washes with RPMI-1640 medium. The cells were then cultured in RPMI-1640 medium supplemented with 10% fetal bovine serum and quantified. These cells were seeded into either 96-well plate at a density of 2 × 10^5^ cells per well or into six-well plates at a density of 2 ×10^6^ cells per well. After incubating for two hours, suspended cells were removed, leaving behind adherent PMs.

Female C57BL/6 and BALB/c mice aged six to eight weeks old were obtained from Beijing Vital River Laboratory Animal Technology Company while *Nlrp3*^-/-^ and *Sting*^-/-^ mice were acquired from The Jackson Laboratory. All animals used in this study were carefully maintained under specific pathogen-free conditions. Ethical approval for all animal experiments was obtained from the Institutional Animal Care and Use Committee of Sichuan University located in Chengdu, Sichuan Province, China, following relevant guidelines established by animal associations.

### Tumor models

For the establishment of the CT26 peritoneal tumor model, female BALB/c mice were intraperitoneally inoculated with a total of 2 × 10^5^ CT26 cells per mouse. In the B16-F10 experimental lung metastasis or B16-F10 peritoneal tumor models, female C57BL/6 mice received intravenous or intraperitoneal injections of a total of 2 × 10^5^ B16-F10 cells per mouse, respectively. Chitosan (9012-76-4, Sigma‒Aldrich), NAG (7512-17-6, Sigma‒Aldrich), or vehicle was administered to the mice. Mice received intraperitoneal injections of chitosan (50, or 100 mg/kg), NAG (1 M solution, 100 μL per mice), or vehicle control alone, with each treatment administered every 3 days for a total of five doses, as specified by the experimental design (Supplemental Figure 1A). After inoculation for 15 days, the mice were sacrificed and evaluated for tumor growth in CT26 and B16-F10 peritoneal models as well as lung metastasis in B16-F10 lung metastasis model. Single-cell suspension from tumor microenvironment (TME) was prepared and subjected to flow cytometry analysis. To monitor tumor progression in the CT26 intraperitoneal tumor model, bioluminescent in vivo imaging was performed at 3- to 4-day intervals using D-luciferin (15 mg/mL, 200 μL per mouse) administered via intraperitoneal injection 5 minutes prior to imaging. This longitudinal tracking approach enabled real-time quantification of tumor burden through luciferase-driven bioluminescent signal acquisition. All animal experiments have been approved by the Institutional Animal Care and Use Committee of Sichuan University (Chengdu, Sichuan, China).

### Flow cytometry analyses of the tumor immune microenvironment

The mice were euthanized to obtain tumor tissues and lung specimens containing tumor nodules for subsequent processing. In a concise summary of the procedure, the collected tissues were finely minced while maintaining low temperatures, followed by digestion in a collagenase buffer solution consisting of 1 mg/mL collagenase I (Gibco), 0.5 mg/mL collagenase IV (Gibco), and 40 U/mL DNase I (Sigma), all dissolved in DMEM (Gibco, USA). The resulting tissue suspensions were incubated at 37 °C for 1 hour with periodic agitation. Subsequently, the suspensions were filtered through a 70 μm nylon mesh and centrifuged at 400 × g for 5 minutes. The resulting cellular pellets were resuspended in 100 μL of PBS. Single cells thus prepared underwent staining with the designated antibodies at a temperature of 4 °C for a duration of 30 minutes, followed by two rounds of washing with 2 mL of PBS. The samples were subjected to analysis utilizing a BD Fortessa flow cytometer, with data analysis conducted using Novoexpress software. The antibodies utilized for flow cytometry analyses, comprising PerCP-conjugated anti-CD45 antibody, FITC/APC/PerCP-conjugated anti-CD11b antibody, FITC/APC/PerCP-conjugated anti-CD11c antibody, BV421/PerCP-conjugated anti-CD3 antibody, APC-conjugated anti-CD4 antibody, FITC-conjugated anti-CD8 antibody, PE-conjugated anti-CD69 antibody, PE/APC-conjugated anti-F4/80 antibody, PE/APC-conjugated anti-CD206 antibody, PE-conjugated anti-Ly6G antibody, FITC-conjugated anti-Ly6C antibody, PE-CY7-conjugated anti-Arg-1 antibody, BV510-conjugated anti-TNF-α antibody were procured from BD Biosciences Company.

### Transfection assays of macrophages

To investigate chitosan-mediated immune responses and elucidate specific mechanisms, primary peritoneal macrophages were isolated. The Lipo2000 transfection kit was utilized for the efficient delivery of chitosan, NAG, GAM, glucose, and sucrose into macrophages. For preparation of the transfection reagents, a sugar solution tailored to the experimental concentration was prepared in Opti-MEM medium. Additionally, 2 μL of Lipofectamine 2000 transfection reagent was added to 100 μL of Opti-MEM medium. Subsequently, gentle mixing combined 100 μL of the sugar solution with 100 μL of the transfection solution followed by a gradual mixing process and a subsequent incubation period at room temperature for 30 minutes.

Peritoneal macrophages (PMs) were pre-treated with 1 μg/mL LPS (Sigma‒Aldrich) for 4 hours, followed by three washes with RPMI-1640 medium to remove any residual serum. Subsequently, the prepared transfection mixture was administered to the cells at a dosage of 180 μL of RPMI-1640 medium and 20 μL of the mixture per well in 96-well plates or 1.8 mL of RPMI-1640 medium and 200 μL of the mixture per well in 6-well plates, followed by a 6-hour incubation.

### Mitochondrial DNA isolation and quantification

The prepared PMs were collected and subjected to two rounds of sterile PBS washing. Subsequently, all cells were evenly distributed into two tubes. One of these tubes was designated for the isolation of mtDNA. The cells were then resuspended with the lysate obtained from the mtDNA isolation kit (Abcam), introduced into a homogenizer under sterile conditions, and disrupted on ice. Following this, centrifugation was performed according to the kit instructions to isolate mtDNA. For assessing mtDNA, the mtCOX1 primer was utilized. The other tube was allocated for total DNA extraction using the DNA Extraction Kits (QIAGEN), employing 18S nuclear DNA primer as a reference. The primer sequences employed in this study are as follows:

mtCOX1-F: 5'-GCCCCAGATATAGCATTCCC-3',

mtCOX1-R: 5'-GTTCATCCTGTTCCTGCTCC-3',

18S-F: 5'-TAGAGGGACAAGTGGCGTTC-3',

18S-R: 5'-CGCTGAGCCAGTCAGTGT-3'.

### Western blot (WB) analyses

For quantification of cytosolic HK2 protein, 300 μL of cell lysis buffer (20 mM HEPES, 10 mM KCl, 1.5 mM MgCl_2_, 1 mM EDTA, 50 mg/mL digitalis saponin, 250 mM sucrose and protease inhibitor) was used to resuspend and lyse the prepared cells on ice for a duration of 10 minutes. Subsequently, the cells were homogenized using a Doones glass homogenizer and then disrupted by passing through a syringe with a 22-gauge needle. The resulting lysates were centrifuged at 13,000 g for a period of 30 minutes to obtain the supernatant containing cytosol devoid of mitochondria. Finally, the protein concentration was determined using the BCA Protein Assay Kit (Beyotime, Beijing, China). Subsequently, 4×Loading Buffer was added at a concentration of 20 μg/10 μL and boiled at 100 ℃ for 10 min. After centrifugation, the samples were stored at -80 ℃ for long-term storage. Additionally, total protein extraction was performed using radioimmunoprecipitation assay (RIPA) buffer (Beyotime, Beijing, China), supplemented with both protease inhibitor cocktail and phosphatase inhibitor. The protein concentration was determined using the BCA Protein Assay Kit (Beyotime, Beijing, China). Protein samples (ranging from 20 to 40 μg per sample) were resolved on polyacrylamide gels containing SDS and subsequently transferred onto nitrocellulose transfer (NC) membranes. Following a 2-hour blocking step with 5% nonfat milk, the membranes were incubated overnight at 4 °C with primary antibodies including IL-1β antibody (Abcam), pro-IL-1β antibody (Abcam), Caspase 1 antibody (Abcam), Cleaved Caspase 1 antibody (Abcam), β-tubulin antibody (Abcam), Hexokinase 2 antibody (Abcam), Tomm20 antibody (Abcam), STING antibody (CST), and cGAS antibody (CST). Afterwards, the membranes were exposed to secondary antibodies [horseradish peroxidase (HRP)-labeled anti-mouse/rabbit IgG] for two hours at room temperature (RT)and visualized using an Odyssey Imager Dual-color infrared imaging system (LI-COR, N.E., USA).

### Mitochondrial transmembrane potential

To evaluate mitochondrial membrane potential, we utilized Rhodamine 123 (RH-123) as a fluorescent probe. The macrophages under investigation were incubated with RH-123 at 37 °C for 30 minutes in the absence of light exposure. Subsequently, the cells were collected, washed, and subjected to flow cytometry analysis. Importantly, it should be noted that the decay rate of RH-123 fluorescence is directly proportional to mitochondrial membrane potential.

### Reactive oxygen species (ROS) detection

To quantitatively measure the levels of reactive oxygen species (ROS) within macrophages, we employed 2',7'-dichlorofluorescin diacetate (H2DCF-DA). Macrophages stimulated with lipopolysaccharide (LPS) were incubated in RPMI-1640 medium supplemented with 25 μM H2DCF-DA at 37 °C while avoiding light exposure for a duration of 45 minutes. Subsequently, the cells underwent two washes. Following this, the cells were stimulated with either chitosan, N-acetylglucosamine (NAG), or adenosine triphosphate (ATP), and absorbance values were continuously recorded over a period of 4 hours. These measurements were performed using an enzyme marker at an excitation wavelength of 485 nm and an emission wavelength of 530 nm.

### Hematein & eosin (HE) staining

The harvested tissues were subsequently fixed in 4% paraformaldehyde for a duration exceeding 72 hours. Following fixation, the tissues underwent a series of procedures, including paraffin embedding, sectioning, dehydration, and hematoxylin and eosin (HE) staining.

### Enzyme-linked immunosorbent assay (ELISA) analyses of cytokines

The levels of IL-1β in vitro were measured using the IL-1 beta Mouse Uncoated ELISA Kit (Thermo Fisher Scientific) following the manufacturer's guidelines.

### CCK-8 proliferation assay

Cell viability and proliferation were assessed using the Cell Counting Kit-8 (CCK-8; Beyotime) according to the manufacturer's protocol. Briefly, cells were seeded into 96-well plates at a density of 6 × 10³ cells/well and incubated under standard culture conditions (37°C, 5% CO₂) for 24 or 48 hours. At each time point, 10 μL of CCK-8 reagent was added to each well, followed by incubation for 2 hours. Absorbance was measured at 450 nm. All experiments included 3 biologically independent wells. Data were normalized to control groups and expressed as relative cell survival rates.

### Annexin V-PI flow cytometry assay

Apoptosis was quantified using an Annexin V-FITC/PI Apoptosis Detection Kit (BD 556547 Annexin V FITC Apoptosis Kit) following the manufacturer's protocol. CT26 pretreated with chitosan or NAG cells were harvested, washed twice with ice-cold PBS, and resuspended in 1× binding buffer at a density of 1 × 10⁶ cells/mL. Subsequently, 100 μL of cell suspension was incubated with 5 μL Annexin V-FITC and 5 μL propidium iodide (PI) for 15 minutes at room temperature in the dark. Stained cells were immediately analyzed using a BD Fortessa flow cytometer. Data were processed using Novoexpress software.

### Hexokinase II VDAC binding domain peptide (HKVBD)

With reference to sequences from previous literature, TAT-fused HKVBD (MIASHLLAYFFTELN(β-Ala) GYGRKKRRQRRRG) and scramble peptide (ATAFLMEYNSHLFIL(β-Ala) GYGRKKRRQRRRG) were synthesized by GL Biochem (Shanghai) Ltd.

### Mitochondrial MitoTracker™ staining

Mitochondrial morphology and mass were assessed using MitoTracker® Red CMXRos (Invitrogen, Cat# M7512). Cells were seeded onto glass-bottom dishes and incubated with 100 nM MitoTracker Red CMXRos (prepared in serum-free medium) for 30 minutes at 37°C under 5% CO₂ in the dark. Following staining, cells were washed three times with warm PBS to remove excess dye and fixed with 4% paraformaldehyde (PFA) for 15 minutes at room temperature. Nuclei were counterstained with DAPI (1 μg/mL, 5 minutes). Fluorescence images were acquired using a confocal laser scanning microscope (Zeiss LSM 880).

### Statistical analyses

The experimental data were statistically analyzed and statistical graphs were generated using GraphPad Prism 8. One-way analysis of variance (ANOVA), two-way ANOVA, or two-sided Student's t-test was employed as appropriate for data analysis. Survival curve analyses were performed using the log-rank test. Mean values accompanied by their respective standard deviations (SD) are presented as results. Statistical differences were indicated as *p < 0.05, **p < 0.01, ***p < 0.001, ****p < 0.0001, with p < 0.05 considered significant according to predetermined criteria.

Other details and additional experimental procedures can be found in the online supplemental methods.

## Supplementary Material

Supplementary figures.
