# Supplementary material for: Chitosan activates NLRP3 inflammasome and cGAS-STING to suppress cancer progression through hexokinase 2 dissociation and mitochondrial dysfunction
Source: Theranostics. 2025 Jul 25;15(16):8473–87. doi: 10.7150/thno.112009 (PMC12374619; doi:10.7150/thno.112009)
Supplement: Supplementary file 1 — Supplementary figures. [file thnov15p8473s1.pdf]

Supplementary Materials

Supplementary materials include 5 Supplementary Figures

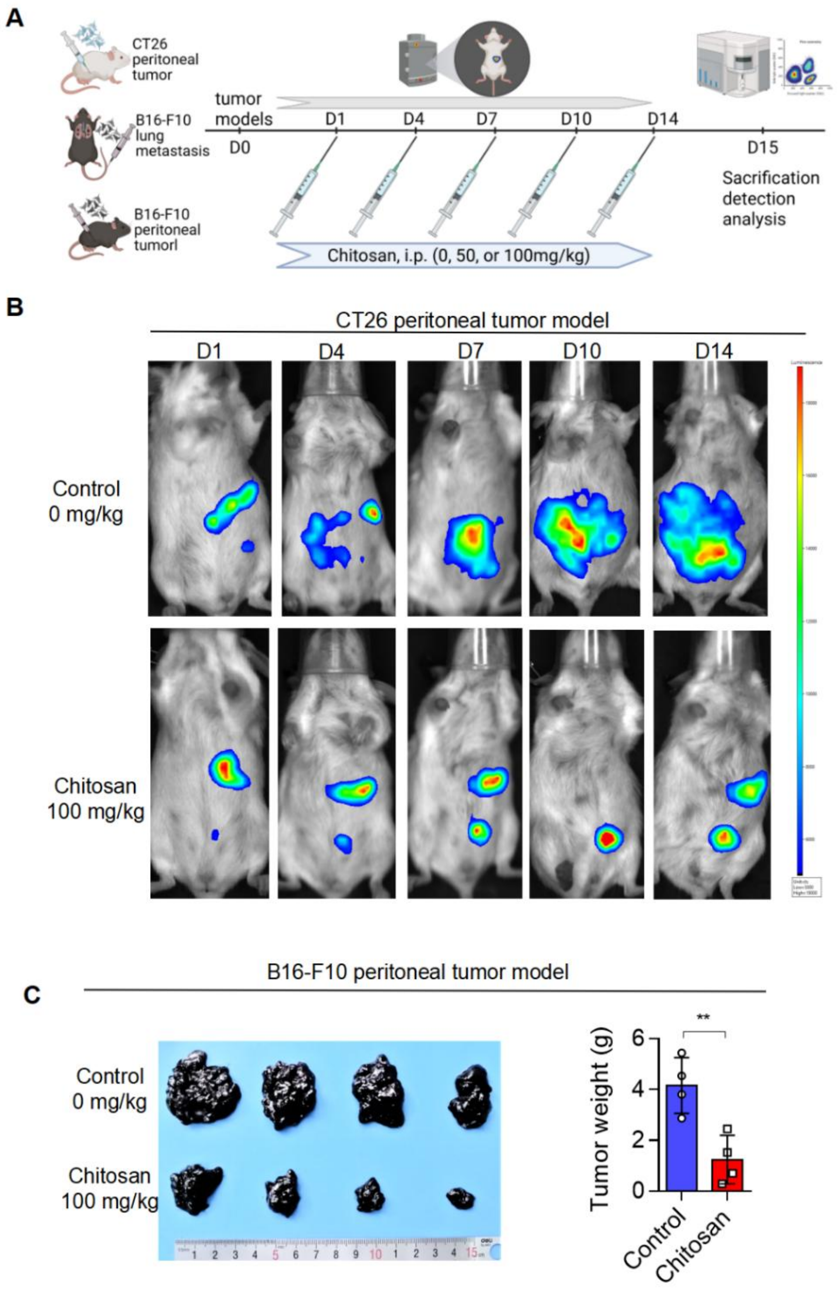

Supplemental Figure S1. Chitosan suppresses tumor growth.

(A) Schematic representation illustrating in vivo experimental design.

(B) Chitosan suppresses tumor growth in CT26 peritoneal tumor model. After receiving isoflurane gas anesthesia, each mouse was injected intraperitoneally with 200 μl of D-luciferin (15 mg/mL)

and 5 min later bioluminescence imaging was performed. Representative images of visualized CT26 peritoneal tumor development on bioluminescence imaging.

(C) B16-F10 cells (2×10^5) were transplanted into the peritoneal cavity of mice to establish B16-F10 peritoneal tumor model (n = 4 mice). Mice were intraperitoneally administrated with 0 or 100 mg/kg chitosan and sacrificed on day 15. Then peritoneal tumors were dissected and gross images were showed (C, left). Tumor weight (C, right) was measured (n = 4 mice).

Data are represented as mean±SD. Statistical significance in (C) was determined by a two-sided unpaired t-test. ** $p < 0.01$.

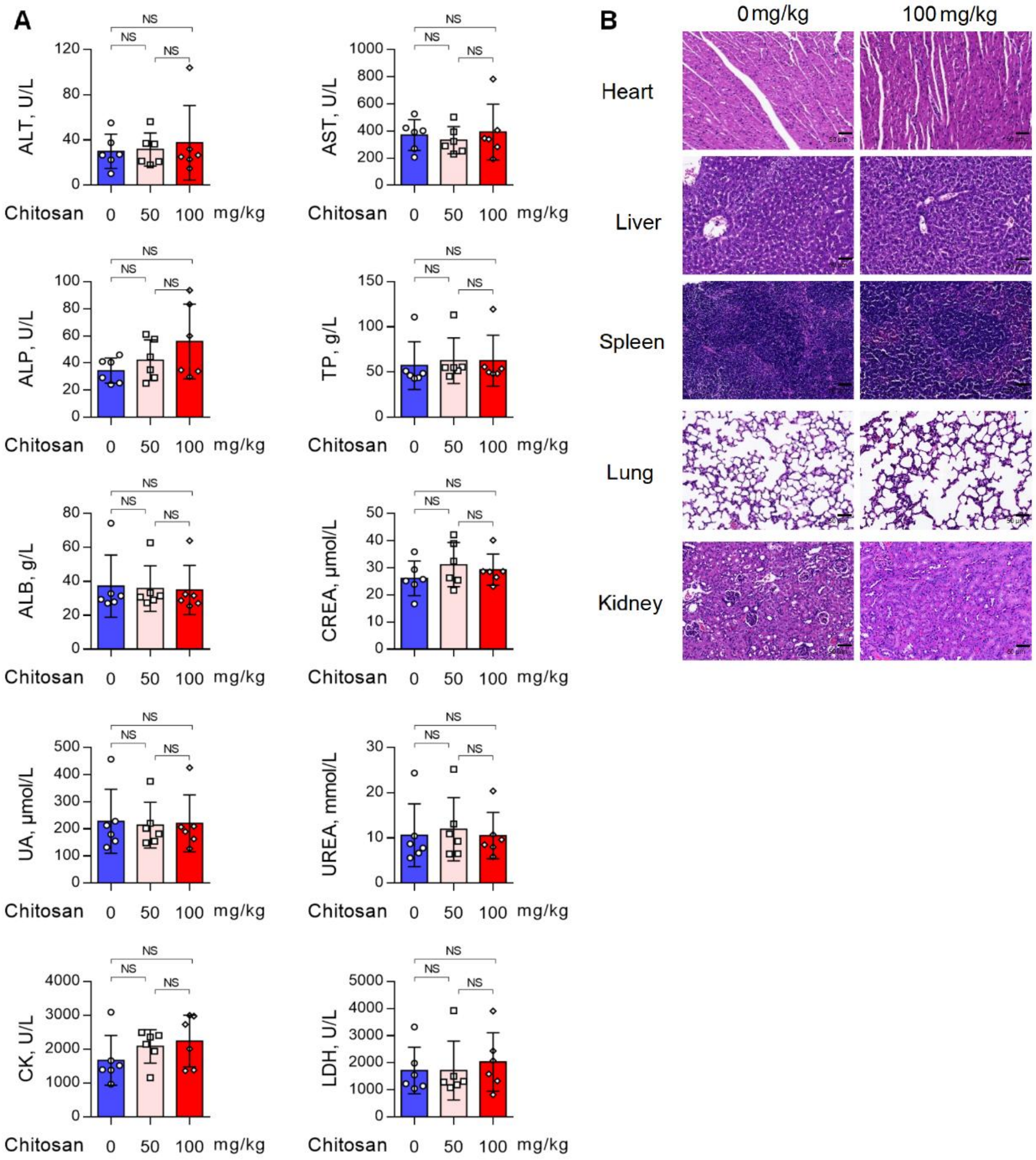

Supplemental Figure S2. Toxicity assessment of chitosan in B16-F10 peritoneal tumor model.

(A-B) B16-F10 cells (2×10^5) were transplanted into the peritoneal cavity of mice to establish B16-F10 peritoneal tumor model (n = 6 mice). Mice were intraperitoneally administrated with 0, 50 mg/kg or 100 mg/kg chitosan and sacrificed on day 15. Blood was collected from the mice's eyeballs, and serum was subsequently obtained. The detection of biochemical indicators, including ALT, AST, ALP, TP, ALB, CREA, UA, UREA, CK and LDH, was completed by fully automatic biochemical analyzer from Mindray (A). Then the heart, liver, spleen, lung, and kidney were dissected and H&E staining were conducted (B). Scale bars represent 2 mm. Data are represented as mean ± SD. Statistical significance in (A) was determined by one-way ANOVA. NS, not

significant.

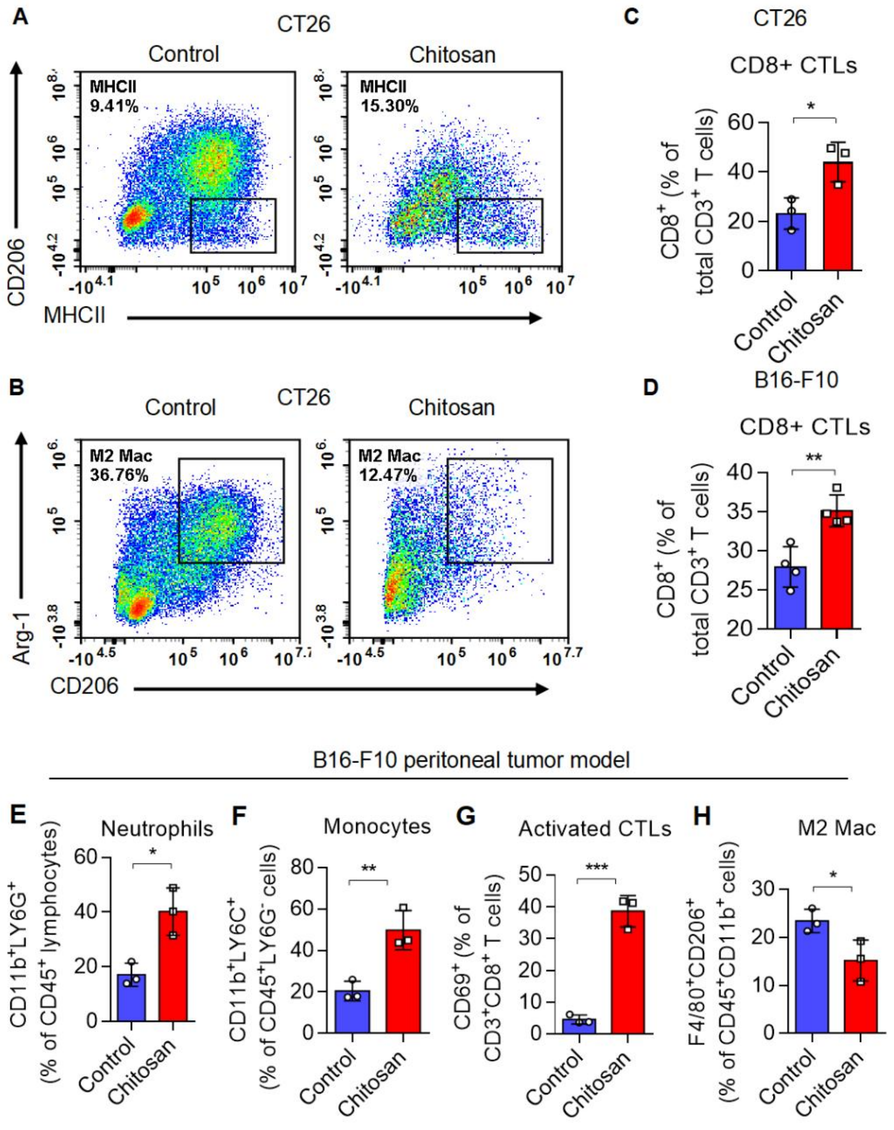

Supplemental Figure S3. Chitosan transforms tumor immune microenvironment and increases infiltration of neutrophils and monocytes.

(A-B) Representative scatterplots of the gated M1 macrophages ($CD45^+$ $CD11b^+$ $F4/80^+$ $MHCII^+$ $CD206^-$) are shown in (A) (n = 4 mice). Representative scatterplots of the gated M2 macrophages ($CD45^+$ $CD11b^+$ $F4/80^+$ $CD206^+$ $Arg\text{-}1^+$) are shown in (B) (n = 4 mice).

(C-D) The percentages of CD8+ CTLs ($CD3^+$ $CD8^+$) in CT26 peritoneal tumor model (C) (n = 3 mice) and B16-F10 experimental pulmonary metastasis models (D) are quantified (n = 4 mice).

(E-H) The single-cell suspension of B16-F10 peritoneal tumors was prepared and subjected to FCM analysis. The percentages of neutrophils ($CD45^+$ $CD11b^+$ $LY6G^+$) (E), monocytes ($CD45^+$

$CD11b^+$ $LY6G^-$ $LY6C^+$) (F), activated CTLs ($CD3^+$ $CD8^+$ $CD69^+$) (G), and M2 Macrophages ($CD45^+$ $CD11b^+$ $F4/80^+$ $CD206^+$) (H) are quantified (n = 3 mice).

Data are represented as mean ± SD. Statistical significance in (C, D, E, F, G, H) was determined by a two-sided unpaired t-test. * $p < 0.05$, ** $p < 0.01$, *** $p < 0.001$.

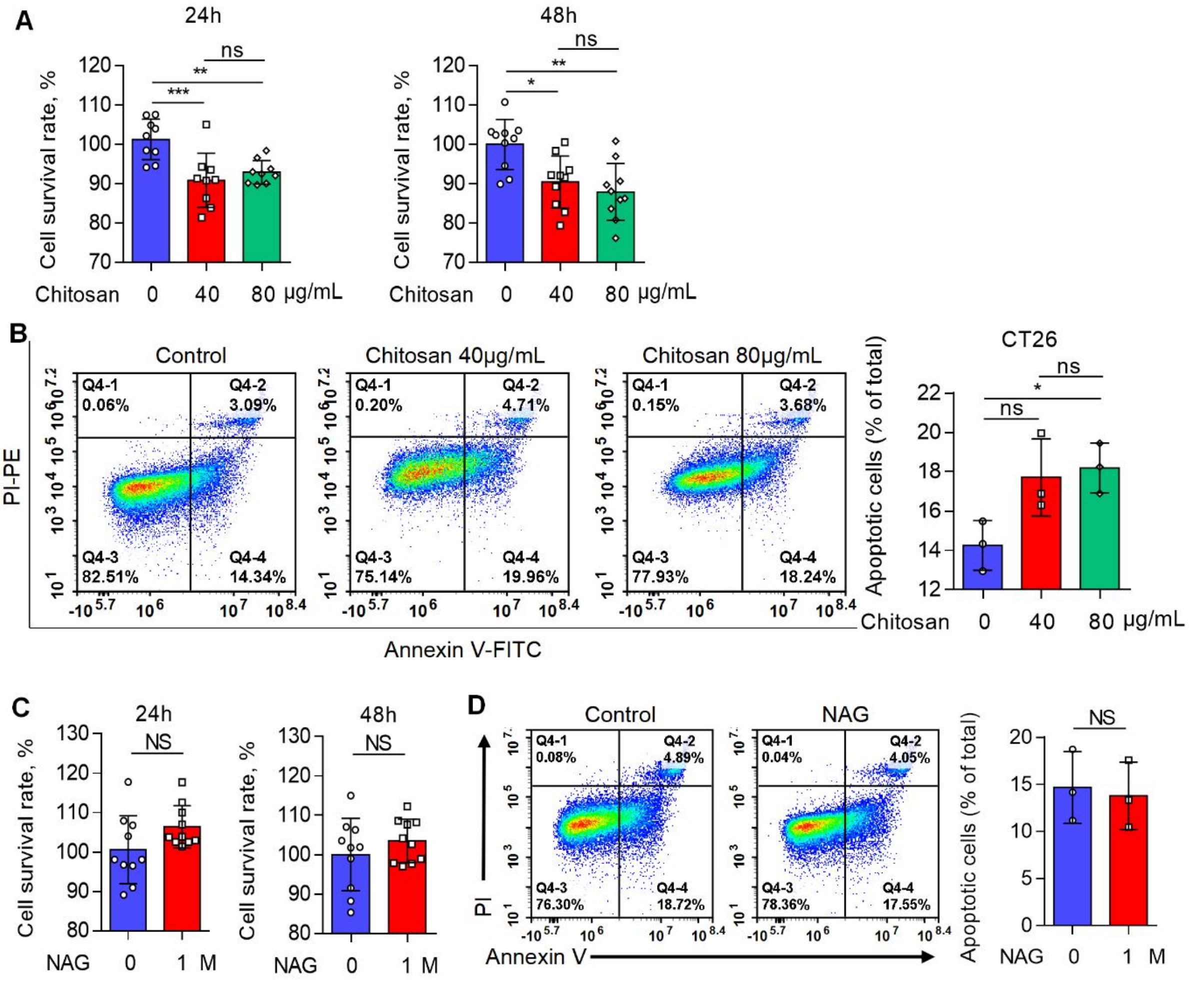

Supplemental Figure S4. Direct effects of chitosan and NAG on CT26 tumor cells

(A-B) Chitosan inhibits CT26 cell proliferation and promotes apoptosis. CCK-8 cell proliferation assay (A) and Annexin V/PI double-staining FCM assay (B) were respectively used to assess the cell proliferation and apoptosis status of CT26 cells after treatment with 0, 40, and 80μg/mL chitosan.

(C-D) NAG has no significant toxic effects on CT26 tumor cells. CCK-8 cell proliferation assay (C) and Annexin V/PI double-staining FCM assay (C) were respectively used to assess the cell proliferation and apoptosis status of CT26 cells after treatment with 0, and 1 M NAG. Data are represented as mean ± SD. Statistical significance in (A-B) was determined by one-way ANOVA. Statistical significance in (C) was determined by a two-sided unpaired t-test. NS, not significant. * $p < 0.05$, ** $p < 0.01$, *** $p < 0.001$.

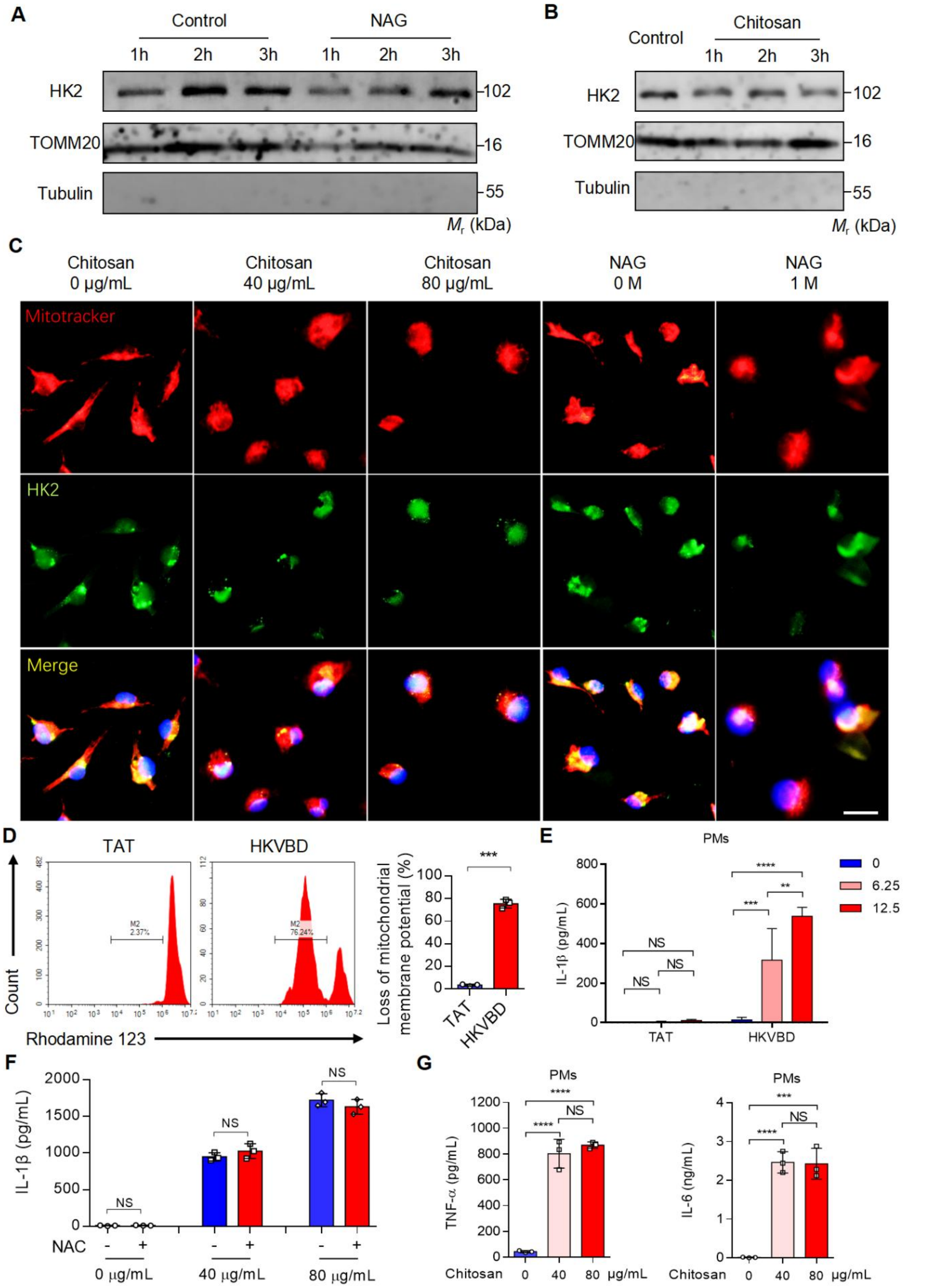

Supplemental Figure S5. Chitosan activates the NLRP3 inflammasome through the dissociation of mitochondrial HK2 and induction of mitochondrial dysfunction.

(A-B) Chitosan activates NLRP3 inflammasome through the dissociation of mitochondrial HK2. LPS-pretreated PMs from WT mice were stimulated with NAG (1 M) (A) or chitosan (80 μg/mL) (B) for indicated time. Then the mitochondrial protein was extracted and HK2 levels were detected by WB. TOMM20 (Translocase of Outer Mitochondrial Membrane 20) was used as a mitochondrial marker. Tubulin was used as a cytoplasmic internal reference protein.

(C) Chitosan induces mitochondrial dysfunction of PMs. LPS-pretreated PMs were stimulated with 0, 40, 80 μg/mL chitosan or 0, 1M NAG. Mitotracker staining was used to assess mitochondrial function and morphology. Association of HK2 with mitochondria was visualized.

(D) LPS-pretreated PMs were stimulated with 0 μM HKVBD (TAT) or 25 μM HKVBD. Mitochondrial membrane potential was detected by Rhodamine 123 (RH123) FCM analysis (n = 3 biologically independent samples).

(E) LPS-pretreated PMs were stimulated with control (TAT) or HKVBD of indicated concentrations. The IL-1β levels in the supernatants were detected by ELISA (n = 3 biologically independent samples).

(F) ROS inhibitor cannot reduce the increased IL-1β levels induced by chitosan. ROS inhibitor, NAC, was used to treat 0, 40, 80μg/mL chitosan stimulated cells. The IL-1β levels in the supernatants were detected by ELISA (n = 3 biologically independent samples).

(G) Chitosan promotes the secretion of TNF-α and IL-6 in PMs. LPS-pretreated PMs from WT mice were stimulated with 0, 40 or 80 μg/mL chitosan for 6 hours. Then the TNF-α and IL-6 levels in the supernatants were detected by ELISA (n = 3 biologically independent samples).

Data are represented as mean ± SD. Statistical significance in (E-G) was determined by one-way ANOVA. NS, not significant. ** $p < 0.01$, *** $p < 0.001$, **** $p < 0.0001$.
